# Evaluating Factors for Prophylactic Feeding Tube Placement in Gastroesophageal Cancer Patients Undergoing Chemoradiotherapy

**DOI:** 10.3389/fonc.2017.00235

**Published:** 2017-09-27

**Authors:** Vivek Verma, Pamela K. Allen, Steven H. Lin

**Affiliations:** ^1^Department of Radiation Oncology, University of Nebraska Medical Center, Omaha, NE, United States; ^2^Department of Radiation Oncology, University of Texas M.D. Anderson Cancer Center, Houston, TX, United States

**Keywords:** esophageal cancer, gastric tube, nutrition, chemotherapy, radiation therapy

## Abstract

**Purpose:**

Though better studied in head/neck cancers, there are currently no studies on timing of feeding tube (FT) placement in patients with gastroesophageal cancer. This study sought to discern characteristics of patients who used versus did not use a prophylactic FT (pFT), and also analyzed factors associated with placement of FTs during chemoradiotherapy (CRT).

**Methods/materials:**

From 1998 to 2013, 1,329 patients underwent neoadjuvant CRT, of which 323 received an FT. Patients for whom FTs were placed prior to treatment due to tumor occlusion or substantial weight loss (*n* = 130), and those with FTs placed following treatment (*n* = 43) were excluded. One hundred patients had pFTs placed, and 50 underwent placement during CRT. The following was collected for each patient: demographic/patient information, oncologic/treatment characteristics, and CRT tolerance.

**Results:**

No significant differences were found in any parameter between cohorts that used (*n* = 66) versus did not use a pFT (*n* = 34); on univariate and multivariate analyses, no pretreatment characteristic associated with using a pFT. When compared with patients who used a pFT (*n* = 66), those who required an FT during CRT (*n* = 50) had lower body mass index (*p* = 0.045), underwent higher-dose radiotherapy (*p* = 0.003), and received induction chemotherapy (*p* = 0.031). On multivariate analysis, receipt of induction chemotherapy and greater weight loss and esophagitis during treatment were associated with placement of FTs during CRT (*p* < 0.05).

**Conclusion:**

Of our cohort who received pFTs, there were no clinical factors that predicted for their use. Patients must be closely monitored for weight loss and esophagitis when receiving CRT in order to intervene prior to further worsening of toxicities.

## Introduction

Feeding tubes (FTs) can be used for nutritional support in cancer patients and are either placed prophylactically or as a response to toxicities during or after oncologic therapy. Though most commonly used in head and neck cancers, timing of use—especially prophylactically—is controversial and without consensus at present (1–3). In comparison, FTs are inserted much less frequently for gastroesophageal (GE) neoplasms. Incidences have been reported as high as 63% (4), but also as low as 8% (5); however, absolute numbers remain low. Nevertheless, poor nutritional status can affect subsequent treatment options and outcomes including those of surgery (6).

No evidence has been published to date examining factors associated with FT insertion at various time points, as well as evaluating prophylactic placement of FTs in GE cancers. As such, the National Comprehensive Cancer Network insinuates that FT placement be considered on a case-by-case basis, especially in conditions of low caloric intake and/or esophageal obstruction (7).

In this study, we sought to examine the population of GE cancers that received FTs, treated at a high-volume, tertiary care academic medical center. Specifically, our study had two principal goals. First, because prophylactic FTs (pFTs) may or may not be utilized, we aimed to ascertain factors associated with needing to use a pFT. Second, a comparison of factors was made between patients who used a pFT and those who required an FT during radiotherapy (RT), in efforts to delineate a high-risk population that could benefit from aggressive supportive care and possibly a lower threshold for FT placement during RT.

## Materials and Methods

### Patient Population

**Figure 1:**
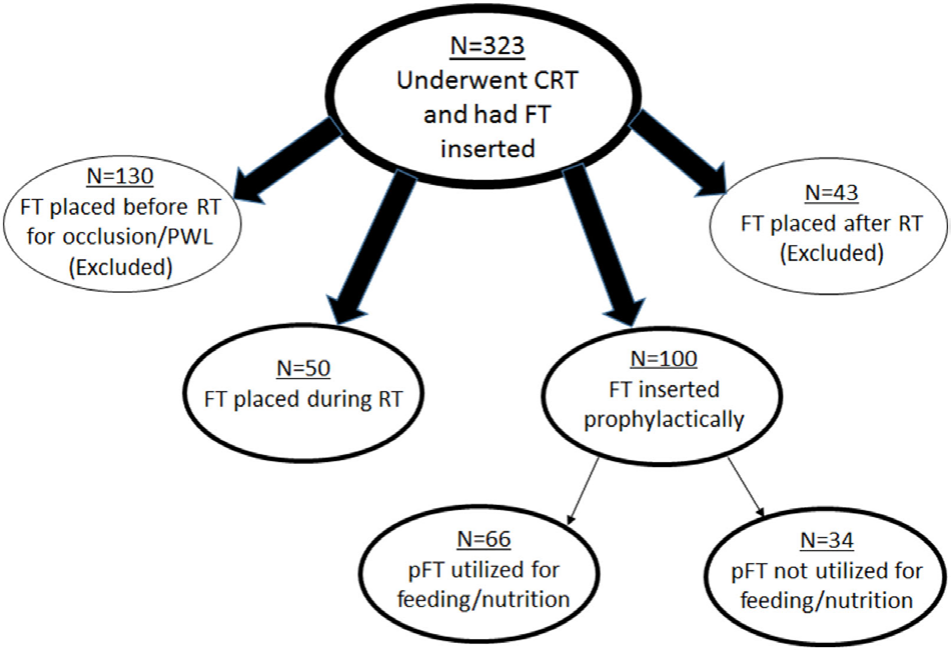
Schematic representation of patient subgroups in this study. CRT, chemoradiotherapy; FT, feeding tube; RT, radiotherapy; PWL, profound weight loss; pFT, prophylactic feeding tube.

This single-institutional analysis examined 1,329 patients with cancer of the esophagus or GE junction treated with concurrent chemoradiotherapy (CRT) as part of neoadjuvant therapy (1998–2013). Of these patients, the analyzed population consisted of those who underwent FT placement, which numbered 323 patients. Each patient provided informed consent to place an FT if recommended. Figure 1 displays patient subgroups. Exclusion criteria for this study included those patients who underwent FT insertion after RT (*n* = 43); though many of these were from CRT-related toxicities, FTs in many others were placed in anticipation of surgery and not directly as a result of toxicities. Similarly, 130 patients had FT placed prior to RT specifically owing to partial or complete luminal occlusion from the malignancy and/or with profound weight loss (PWL) (greater than 10% weight loss in past 3–6 months). Because this constituted a heterogeneous patient population for the purposes of our analysis, they were additionally excluded.

These criteria left 150 patients for further investigation. Of these patients, 50 had placement during RT, and 100 were placed prophylactically. Reasons for FT placement during RT included odynophagia, dysphagia, decreased appetite, and nausea/vomiting. pFT placement was performed at the clinician’s discretion, based on clinical concern but not due to any of the aforementioned rationale for FT placement. Whether FTs were utilized for nutrition (defined as at least one administration of enteral feeding) was then recorded; 66 patients used the FT and 34 did not.

Two comparisons between patient subgroups were made in this study: (1) factors associated with use of a pFT (*n* = 66) versus lack thereof (*n* = 34) and (2) characteristics between patients who used a pFT (*n* = 66) versus those who had FT insertion during RT (*n* = 50). For each patient in each subgroup, many parameters were collected, broadly including patient/demographic information, tumor and treatment characteristics, and intra-CRT tolerance.

### Oncologic Therapy

Radiotherapy was administered in three possible modalities: three-dimensional conformal RT, intensity-modulated RT, or proton beam RT. Briefly, for each modality, patients were immobilized using custom molds and simulated in the supine position with four-dimensional computed tomography simulation. Positron emission tomography fusion aided in target volume definition. In general, doses consisted of 41.4–50.4 Gy and were delivered with concurrent chemotherapy, which consisted of various regimens over time and at the medical oncologist’s discretion. Patients were seen weekly by the medical and radiation oncologists on treatment, with reassessment of toxicities and potential intervention at each visit. Surgery was performed several weeks after cessation of CRT and most commonly was transthoracic esophagectomy.

### Statistical Analysis

Data analysis was performed using Stata/MP14 statistical software (College Station, TX, USA). The Wilcoxon rank-sum and Fisher’s exact tests were used to compare the factors associated with use and lack of use of a pFT, as well as to compare the characteristics of patients who used a pFT against those whom required an FT placed during treatment. Univariate and multivariate logistic regression were performed in order to examine variables associated with placement of FTs in various circumstances. *p*-values <0.05 were considered to be statistically significant. Statistical tests were based on a two-sided significance level.

## Results

Table 1 displays characteristics of patients who did (*n* = 66) versus did not utilize (*n* = 34) their pFT. There were no statistically significant differences between groups in any parameter analyzed, including performance status, body mass index (BMI) at diagnosis, tumor length/stage, symptoms at diagnosis, target volume size, or RT dose. Univariate analysis, performed in order to examine variables associated with use of a pFT, did not show correlation with any examined variable (Table 2). On multivariate analysis, the only factor associated with use of a pFT was weight loss during treatment [continuous variable; odds ratio (OR) 1.16, 95% confidence interval (CI) 1.01–1.32, *p* = 0.03]. Hence, no pretreatment parameter was found to be reliably associated with use of a pFT.

**Table 1:** Clinical characteristics of patients who used versus did not use a pFT.

Parameter	All patients with pFT (*n* = 100)	Did not use pFT (*n* = 34)	Used pFT (*n* = 66)	*p*-values
Median age at diagnosis (years) (range)	64 (21–84)	65 (29–84)	63.5 (21–84)	0.951
Gender				
Male	80 (80%)	29 (85%)	51 (77%)	0.434
Female	20 (20%)	5 (15%)	15 (23%)	
Median body mass index (kg/m^2^) (range)	26.8 (16.5–43.0)	27.3 (19.9–35.5)	25.5 (16.5–43.0)	0.334
Karnofsky performance status				
60	2 (2%)	1 (3%)	1 (2%)	0.569
70	6 (6%)	1 (3%)	5 (8%)
80	44 (44%)	16 (47%)	28 (42%)
90	40 (40%)	15 (44%)	25 (38%)
100	8 (8%)	1 (3%)	7 (11%)
Location				
Upper	17 (17%)	8 (24%)	9 (14%)	0.171
Middle	7 (7%)	4 (12%)	3 (5%)
Lower	76 (76%)	22 (65%)	54 (82%)
Median tumor length (cm) (range)	6 (1–15)	7 (2–13)	5 (1–15)	0.065
AJCC clinical T stage				
T1	1 (1%)	0 (0%)	1 (2%)	0.651
T2	9 (9%)	4 (12%)	5 (8%)
T3	83 (83%)	29 (85%)	54 (82%)
T4	7 (7%)	1 (3%)	6 (9%)
AJCC clinical N stage				
N0	32 (32%)	10 (29%)	22 (33%)	0.822
N1	68 (68%)	24 (71%)	44 (67%)
Receipt of induction chemotherapy				
No	47 (47%)	17 (50%)	30 (45%)	0.679
Yes	53 (53%)	17 (50%)	36 (54%)
Dysphagia symptoms at diagnosis				
No	28 (28%)	7 (21%)	21 (32%)	0.347
Yes	72 (72%)	27 (79%)	45 (68%)
Odynophagia symptoms at diagnosis				
No	81 (81%)	28 (82%)	53 (80%)	0.999
Yes	19 (19%)	6 (18%)	13 (20%)
Median weight loss at diagnosis (kg) (range)	3.0 (0–27)	1.5 (0–20)	3 (0–27)	0.238
Median total RT dose (Gy) (range)	50.4 (16.2–66)	50.4 (16.2–66)	50.4 (32.4–62.8)	0.098
Median PTV volume (cm^3^) (range)	800 (45–3,080)	798 (210–2,168)	801 (45–3,080)	0.743
RT modality				
3DCRT	47 (47%)	16 (47%)	31 (47%)	0.835
IMRT	47 (47%)	17 (50%)	30 (45%)	
PBT	6 (6%)	1 (3%)	5 (8%)	
Median weight loss during RT (kg) (range)	3.9 (0–16.5)	3.0 (0–15.0)	4.5 (0–16.5)	0.143

*pFT, prophylactic feeding tube; AJCC, American Joint Commission on Cancer; PTV, planning target volume; RT, radiotherapy; 3DCRT, three-dimensional conformal radiotherapy; IMRT, intensity-modulated radiotherapy; PBT, proton beam therapy*.

**Table 2:** Univariate analysis of factors associated with use of a prophylactic feeding tube.

Parameter	Odds ratio	95% CI	*p*-values
Median age at diagnosis			
Continuous variable	1	0.97–1.04	0.876
Gender			
Male versus female	0.59	0.19–1.78	0.346
Body mass index			
Continuous variable	0.98	0.89–1.07	0.602
Karnofsky performance status			
Continuous variable	1.01	0.96–1.06	0.668
Location			
Middle versus upper	0.67	0.11–3.93	0.654
Lower versus upper	2.18	0.75–6.38	0.154
Tumor length			
Continuous variable	0.86	0.72–1.01	0.072
AJCC clinical T stage			
T3/4 versus T1/2	1.33	0.35–5.09	0.674
AJCC clinical N stage			
N1 versus N0	0.83	0.34–2.05	0.691
Receipt of induction chemotherapy			
Yes versus no	1.2	0.52–2.75	0.666
Dysphagia symptoms at diagnosis			
Yes versus no	0.56	0.21–1.48	0.24
Odynophagia symptoms at diagnosis			
Yes versus no	1.14	0.39–3.34	0.805
Weight loss at diagnosis			
Continuous variable	1.05	0.98–1.14	0.178
Total RT dose			
Continuous variable	0.96	0.90–1.02	0.199
PTV volume			
Continuous variable	1	0.99–1.01	0.613
RT modality			
IMRT versus 3DCRT	0.91	0.39–2.13	0.829
PBT versus 3DCRT	2.58	0.28–24.00	0.405
Weight loss during RT			
Continuous variable	1.1	0.98–1.23	0.117

*CI, confidence interval; AJCC, American Joint Commission on Cancer; RT, radiotherapy; PTV, planning target volume; IMRT, intensity-modulated radiotherapy; 3DCRT, three-dimensional conformal radiotherapy; PBT, proton beam therapy*.

Next, attention was shifted to compare patients who used a pFT (*n* = 66) versus those who had an FT placed during CRT (*n* = 50). Results of this comparison are shown in Table 3. Of note, lower BMI (median 25.5 versus 23.9, *p* = 0.045) and higher-dose RT (median 50.4 versus 50.4, *p* = 0.003) corresponded to FT placement during CRT. This cohort that underwent FT insertion during CRT experienced greater weight loss on treatment (mean 7.6 versus 4.9%, *p* = 0.002) and higher toxicity due to both dysphagia (*p* < 0.001) and esophagitis (*p* < 0.001). These patients were also more likely to have missed RT treatments, as three patients missed seven or more sessions. Table 4 displays results of univariate analysis of parameters associated with placement of an FT during RT: induction chemotherapy, RT dose, size of the planning target volume, and toxicities during RT (weight loss, dysphagia, esophagitis). Multivariate analysis revealed that patients undergoing induction chemotherapy were more likely to undergo FT placement during RT (OR 2.46, 95% CI 1.09–5.57, *p* = 0.031). Other factors included patients suffering greater weight loss during the treatment course (OR 0.31, 95% CI 0.11–0.90, *p* = 0.031) as well as esophagitis (OR 0.20, 95% CI 0.06–0.61, *p* = 0.005).

**Table 3:** Clinical characteristics of patients who used a pFT versus those who received one during RT.

Parameter	Used pFT (*n* = 66)	FT during RT (*n* = 50)	*p*-values
Median age at diagnosis (years) (range)	63.5 (21–84)	63.5 (22–91)	0.863
Gender			0.999
Male	51 (77%)	39 (78%)
Female	15 (23%)	11 (22%)
Median body mass index (kg/m^2^) (range)	25.5 (16.5–43.0)	23.9 (17.4–39.6)	**0.045**
Karnofsky performance status			
60	1 (2%)	0 (0%)	0.104
70	5 (8%)	6 (12%)
80	28 (42%)	23 (46%)
90	25 (38%)	21 (42%)
100	7 (11%)	0 (0%)
Location			
Upper	9 (14%)	5 (10%)	0.43
Middle	3 (5%)	5 (10%)
Lower	54 (82%)	40 (80%)
Median tumor length (cm) (range)	5 (1–15)	5 (1–10)	0.351
AJCC clinical T stage			
T1	1 (2%)	1 (2%)	0.861
T2	5 (8%)	6 (12%)
T3	54 (82%)	38 (76%)
T4	6 (9%)	4 (8%)
TX	0 (0%)	1 (2%)
AJCC clinical N stage			
N0	22 (33%)	13 (26%)	0.54
N1	44 (67%)	36 (72%)
NX	0 (0%)	1 (2%)
Receipt of induction chemotherapy			
No	30 (45%)	32 (64%)	0.061
Yes	36 (54%)	18 (36%)
Dysphagia symptoms at diagnosis			
No	21 (32%)	11 (22%)	0.296
Yes	45 (68%)	39 (78%)
Odynophagia symptoms at diagnosis			
No	53 (80%)	43 (86%)	0.467
Yes	13 (20%)	7 (14%)
Median weight loss at diagnosis (kg) (range)	3.0 (0–27)	6.8 (0–27)	0.074
Median total RT dose (Gy) (range)	50.4 (32.4–62.8)	50.4 (16.2–66)	**0.003**
Median PTV volume (cm^3^) (range)	801 (45–3,080)	683 (118–1,525)	0.106
RT modality			
3DCRT	31 (47%)	19 (38%)	0.535
IMRT	30 (45%)	25 (50%)
PBT	5 (8%)	6 (12%)
Median weight loss during RT (kg) (range)	4.5 (0–16.5)	7.4 (0–18.1)	**0.002**
Grade of weight loss			
0	33 (50%)	9 (18%)	**0.001**
1	25 (37%)	27 (54%)
2	7 (11%)	12 (24%)
3	0 (0%)	2 (4%)
Unknown	1 (2%)	0 (0%)
Grade of dysphagia			
0	18 (27%)	7 (14%)	**<0.001**
1	14 (21%)	3 (6%)
2	21 (32%)	11 (22%)
3	13 (20%)	29 (58%)
Grade of esophagitis			
0	14 (21%)	2 (4%)	**<0.001**
1	7 (11%)	3 (6%)	
2	33 (50%)	9 (18%)	
3	12 (18%)	36 (72%)	

*Statistically significant p-values are in bold*.

*pFT, prophylactic feeding tube; RT, radiotherapy; AJCC, American Joint Commission on Cancer; PTV, planning target volume; 3DCRT, three-dimensional conformal radiotherapy; IMRT, intensity-modulated radiotherapy; PBT, proton beam therapy*.

**Table 4:** Univariate analysis of factors associated with placement of a feeding tube during RT treatments.

Parameter	Odds ratio	95% CI	*p*-values
Median age at diagnosis			
Continuous variable	0.99	0.96–1.02	0.631
Gender			
Male versus female	0.96	0.40–2.32	0.926
Body mass index			
Continuous variable	1.08	1.00–1.18	0.064
Karnofsky performance status			
Continuous variable	1.03	0.98–1.08	0.207
Location			
Middle versus upper	0.33	0.06–2.02	0.232
Lower versus upper	0.75	0.23–2.41	0.629
Tumor length			
Continuous variable	1.13	0.96–1.34	0.152
AJCC clinical T stage			
T3/4 versus T1/2	1.67	0.52–5.31	0.388
AJCC clinical N stage			
N1 versus N0	0.72	0.32–1.63	0.434
Receipt of induction chemotherapy			
Yes versus no	2.13	1.00–4.53	**0.049**
Dysphagia symptoms at diagnosis			
Yes versus no	0.6	0.26–1.41	0.243
Odynophagia symptoms at diagnosis			
Yes versus no	1.51	0.55–4.11	0.423
Weight loss at diagnosis			
Continuous variable	0.95	0.90–1.00	**0.05**
Total RT dose			
Continuous variable	0.93	0.86–1.00	**0.041**
PTV volume			
Continuous variable	1	1.00–1.00	**0.039**
RT modality			
IMRT versus 3DCRT	0.74	0.34–1.60	0.44
PBT versus 3DCRT	0.51	0.14–1.91	0.317
Weight loss during RT			
Continuous variable	0.87	0.80–0.95	**0.003**
Grade of weight loss			
2–3 versus 0–1	0.31	0.11–0.84	**0.022**
Grade of dysphagia			
2–3 versus 0–1	0.27	0.11–0.62	**0.002**
Grade of esophagitis			
2–3 versus 0–1	0.24	0.08–0.69	**0.008**

*Statistically significant p-values are in bold*.

*CI, confidence interval; AJCC, American Joint Commission on Cancer; RT, radiotherapy; PTV, planning target volume; IMRT, intensity-modulated radiotherapy; 3DCRT, three-dimensional conformal radiotherapy; PBT, proton beam therapy*.

## Discussion

Optimal timing of FT placement in GE cancers is controversial from limited evidence. In our cohort of patients, there were no pretreatment predictors that associated with utilization of a pFT. Hence, because none of the analyzed pretreatment patient, oncologic, or treatment characteristics predicted for utilization of a pFT, these data do not argue in favor of pFT placement. Moreover, receipt of more aggressive therapy (e.g., induction chemotherapy) along with greater symptoms (e.g., weight loss, esophagitis) associated with insertion of FT during RT; hence, select patients must be aggressively monitored for symptoms in order to perform early interventions. Not doing so may risk greater symptomatic suffering and potentially even compromised outcomes from missing RT sessions.

In high-risk patients, both patient-reported and physician-appraised symptoms should be continually re-assessed weekly (at minimum) during treatment, although this may not necessarily mean a lower threshold for FT insertion (other measures such as oral supplements may be considered first). This is important because early intensive nutritional intervention in patients with upper GI cancers results in significantly improved quality of life and body weight (8). Additionally, intensive nutritional support has been shown to decrease the levels of morbidity associated with chemotherapy and the postoperative period (9).

Our data are consistent with those of head and neck cancers; for instance, investigators determined in a report that nearly half of pFTs were not “used” (defined as <2 weeks of feeding) (10). Though the authors did not examine factors that led to “use” of a pFT, our study has not provided justification for pFT insertion in most GE cancers. A notable exception is in cases with tumor obstruction and/or substantial weight loss, which were excluded from our analysis, as these patients often need pFTs—justifiably so—for different circumstances than treatment-related factors alone. This notion may bring forth other issues as to how much weight loss or how much partial obstruction is “substantial” enough to warrant a pFT, in light of other options such as esophageal stenting (11). This may be difficult to test, not only because of the low sample sizes of these patients, but also the large range in clinician/institutional thresholds for pFT insertion.

Limitations to these data are as follows. Though retrospective, there are few prospective data that even detail FT insertion rates; secondary analyses of the many trials examining trimodality therapy are therefore important. The low sample sizes are also noteworthy, but the volume of applicable patients in this study is nearly twice as large as any prospective trial. Our results were not aimed to address factors that are associated with FT or pFT insertion, which would necessitate comparison with the 1,006 patients who did not have FT insertion. Because insertion of (p)FTs is often clinician- and institution-dependent, such results may not be applicable to other institutional policies. Moreover, it is acknowledged that specific reasons for (p)FT placement are also largely institution- and clinician-dependent, limiting applicability to all centers. However, this is a noted limitation facing any study (whether GE cancers or head and neck cancers) examining FTs, and hence may still pertain to practices with somewhat similar reasons for FT placement. Lastly, we excluded 43 patients with post-CRT FT insertion, largely because a proportion undergo placement in anticipation of surgery and not necessarily as a result of toxicities. However, it is still acknowledged that esophagitis peaks 1–2 weeks after CRT, and excluding these patients may miss a proportion of patients with toxicity-related FT placement. This necessitates further research as to which patients are at greatest need for enteral feeding from postoperative complications (12–15).

## Conclusion

In GE cancers, with exception of patients having tumor obstruction and/or PWL, these data do not support insertion of FTs prophylactically prior to CRT. In patients receiving induction chemotherapy, higher-dose RT, and/or having lower pretreatment BMI, early and active symptomatic surveillance is indicated in order to intervene before toxicities worsen.

## Ethics Statement

This study was carried out in accordance with the recommendations of the University of Texas MD Anderson Institutional Review Board and Ethics Committee with written informed consent from all subjects. All subjects gave written informed consent in accordance with the Declaration of Helsinki. Permission was obtained to conduct and publish the study.

## Author Contributions

VV wrote the manuscript, PA analyzed data, SL supervised the study and conceived of its design. All authors read, edited, and approved the final manuscript.

## Disclaimer

This has been presented in part at the 2016 annual meeting of the American Society of Radiation Oncology, September 2016.

## Conflict of Interest Statement

SL has research funding from Elekta, STCube Pharmaceuticals, Peregrine, Bayer, and Roche/Genentech, has served as consultant for AstraZeneca, and received honorarium from US Oncology and ProCure. All other authors declare that the research was conducted in the absence of any commercial or financial relationships that could be construed as a potential conflict of interest.

## References

[1] Koyfman SA Adelstein DJ. Enteral feeding tubes in patients undergoing definitive chemoradiation therapy for head-and-neck cancer: a critical review. Int J Radiat Oncol Biol Phys (2012) 84:581–9. 10.1016/j.ijrobp.2012.03.053 22857885

[2] Verma V Liu J Eschen L Danieley J Spencer C Lewis JS Jr Pre-radiotherapy feeding tube identifies a poor prognostic subset of postoperative p16 positive oropharyngeal carcinoma patients. Radiat Oncol (2015) 10:8. 10.1186/s13014-014-0314-3 25572866 PMC4333178

[3] Verma V Ganti AK Concurrent chemotherapy in older adults with squamous cell head & neck cancer: evidence and management. J Geriatr Oncol (2016) 7:145–53. 10.1016/j.jgo.2016.01.010 26924572

[4] Urba SG Orringer MB Turrisi A Iannettoni M Forastiere A Strawderman M. Randomized trial of preoperative chemoradiation versus surgery alone in patients with locoregional esophageal carcinoma. J Clin Oncol (2001) 19:305–13. 10.1200/JCO.2001.19.2.305 11208820

[5] van Meerten E Muller K Tilanus HW Siersema PD Eijkenboom WM van Dekken H Neoadjuvant concurrent chemoradiation with weekly paclitaxel and carboplatin for patients with oesophageal cancer: a phase II study. Br J Cancer (2006) 94:1389–94. 10.1038/sj.bjc.6603134 16670722 PMC2361286

[6] Nozoe T Kimura Y Ishida M Saeki H Korenaga D Sugimachi K. Correlation of pre-operative nutritional condition with post-operative complications in surgical treatment for oesophageal carcinoma. Eur J Surg Oncol (2002) 28:396–400. 10.1053/ejso.2002.1257 12099649

[7] National Comprehensive Cancer Network. Esophageal and Esophagogastric Junction Cancers. Version 1.2016. (2016). Available from: https://www.nccn.org/professionals/physician_gls/pdf/esophageal.pdf 10.6004/jnccn.2015.0028 25691612

[8] Silvers MA Savva J Huggins CE Truby H Haines T. Potential benefits of early nutritional intervention in adults with upper gastrointestinal cancer: a pilot randomised trial. Support Care Cancer (2014) 22:3035–44. 10.1007/s00520-014-2311-3 24908429

[9] Ligthart-Melis GC Weijs PJ te Boveldt ND Buskermolen S Earthman CP Verheul HM Dietician-delivered intensive nutritional support is associated with a decrease in severe postoperative complications after surgery in patients with esophageal cancer. Dis Esophagus (2013) 26:587–93. 10.1111/dote.12008 23237356

[10] Madhoun MF Blankenship MM Blankenship DM Krempl GA Tierney WM. Prophylactic PEG placement in head and neck cancer: how many feeding tubes are unused (and unnecessary)? World J Gastroenterol (2011) 17:1004–8. 10.3748/wjg.v17.i8.1004 21448351 PMC3057142

[11] Bower MR Martin RC 2nd. Nutritional management during neoadjuvant therapy for esophageal cancer. J Surg Oncol (2009) 100:82–7. 10.1002/jso.21289 19373870

[12] Mariette C De Botton ML Piessen G. Surgery in esophageal and gastric cancer patients: what is the role for nutrition support in your daily practice? Ann Surg Oncol (2012) 19:2128–34. 10.1245/s10434-012-2225-6 22322948

[13] Lin SH Wang L Myles B Thall PF Hofstetter WL Swisher SG Propensity score-based comparison of long-term outcomes with 3-dimensional conformal radiotherapy vs intensity-modulated radiotherapy for esophageal cancer. Int J Radiat Oncol Biol Phys (2012) 84:1078–85. 10.1016/j.ijrobp.2012.02.015 22867894 PMC3923623

[14] Wang J Wei C Tucker SL Myles B Palmer M Hofstetter WL Predictors of postoperative complications after trimodality therapy for esophageal cancer. Int J Radiat Oncol Biol Phys (2013) 86:885–91. 10.1016/j.ijrobp.2013.04.006 23845841 PMC3786201

[15] Juloori A Tucker SL Komaki R Liao Z Correa AM Swisher SG Influence of preoperative radiation field on postoperative leak rates in esophageal cancer patients after trimodality therapy. J Thorac Oncol (2014) 9:534–40. 10.1097/JTO.0000000000000100 24736077 PMC3989552

